# Real-World Hearing Aid Usage Patterns and Smartphone Connectivity

**DOI:** 10.3389/fdgth.2021.722186

**Published:** 2021-08-20

**Authors:** Jeppe Høy Christensen, Gabrielle H. Saunders, Lena Havtorn, Niels H. Pontoppidan

**Affiliations:** ^1^Eriksholm Research Centre, Oticon A/S, Snekkersten, Denmark; ^2^Manchester Centre for Audiology and Deafness, School of Health Sciences, University of Manchester, Manchester, United Kingdom

**Keywords:** hearing aids, smartphone connectivity, usage patterns, acoustic environment, K-means clustering

## Abstract

Data for monitoring individual hearing aid usage has historically been limited to retrospective questionnaires or data logged intrinsically in the hearing aid cumulatively over time (e. g., days or more). This limits the investigation of longitudinal interactions between hearing aid use and environmental or behavioral factors. Recently it has become possible to analyze remotely logged hearing aid data from in-market and smartphone compatible hearing aids. This can provide access to novel insights about individual hearing aid usage patterns and their association to environmental factors. Here, we use remotely logged longitudinal data from 64 hearing aid users to establish basic norms regarding smartphone connectivity (i.e., comparing remotely logged data with cumulative true hearing aid on-time) and to assess whether such data can provide representative information about ecological usage patterns. The remotely logged data consists of minute-by-minute timestamped logs of cumulative hearing aid on-time and characteristics of the momentary acoustic environment. Using K-means clustering, we demonstrate that hourly hearing aid usage patterns (i.e., usage as minutes/hour) across participants are separated by four clusters that account for almost 50% of the day-to-day variation. The clusters indicate that hearing aids are worn either sparsely throughout the day; early morning to afternoon; from noon to late evening; or across the day from morning to late evening. Using linear mixed-effects regression modeling, we document significant associations between daily signal-to-noise, sound intensity, and sound diversity with hearing aid usage. Participants encounter louder, noisier, and more diverse sound environments the longer the hearing aids are worn. Finally, we find that remote logging via smartphones underestimates the daily hearing aid usage with a pooled median of 1.25 h, suggesting an overall connectivity of 85%. The 1.25 h difference is constant across days varying in total hearing aid on-time, and across participants varying in average daily hearing aid-on-time, and it does not depend on the identified patterns of daily hearing aid usage. In sum, remote data logging with hearing aids has high representativeness and face-validity, and can offer ecologically true information about individual usage patterns and the interaction between usage and everyday contexts.

## Introduction

The real world benefit obtained from hearing aids varies considerably across individuals (1). This is thought to be due to individual differences in cognition and working memory (2), variability in hearing aid programming (3, 4), and most relevant to this paper, differences in contextual exposure and needs (5, 6), which can be influenced by both environmental factors (7), individual preferences (8, 9), and listening intentions (6). For example, an individual who needs to hear speech in complex listening environments, such as during a large meeting in a noisy office, will obtain less real world hearing aid benefit than an individual whose listening needs are lower demand such as one-on-one conversations in a quiet room (10).

In the past it has been difficult to assess real world hearing aid use and outcomes because assessments have had to rely on retrospective reports from users combined with limited information collected via intrinsic hearing aid data logging or self-reports. The perceived total use-time is often over-estimated (11–13) or inaccurate depending on hearing aid experience (14, 15). With intrinsic data logging, information about use time, program usage, time spent streaming data, sound pressure level input, listening environment classification, directional microphone settings, and signal-to-noise ratio is stored within the hearing aid (16). However, because space for data storage within the hearing aid is limited, intrinsically-logged data are saved cumulatively over time (17, 18). This not only limits the temporal resolution of the data but also means it is not possible to link patterns of hearing aid usage to specific sound environments and listening conditions.

Ecological momentary assessment (EMA) is another approach that has been used to examine real world hearing aid use and benefit. With EMA, participants describe real world experiences in real time in their own natural environments (19). Recently EMA has been used in several hearing-related studies [see review by Holube et al. (20)]. Most recently it has been used to examine how momentary contextual factors influence subjective ratings of hearing aid outcome (5, 21), differences between listening behaviors of people seeking hearing aids and those who already use them, with a view to predicting who will and will not become a successful hearing aid user (22), and to compare *in-situ* vs. retrospective reports of hearing aid outcome (21, 23). In many hearing-related studies, the participant's EMA reports have been linked in time to an analysis of the sound environment collected by the hearing aids. In some studies, an EMA survey is triggered when a predetermined acoustic environment is encountered (5, 24), while in others, a sound analysis is conducted when a randomly prompted or voluntarily initiated EMA assessment occurs (21, 25). In both instances, there is a direct link between an acoustic analysis of the sound environment and responses to an EMA survey. However, EMA is somewhat intrusive, requiring participants to be willing to answer surveys on multiple occasions during the day. Further, recent work has shown that in certain situations EMA surveys go uncompleted more frequently than in other situations. Specifically, Schinkel-Bielefeld et al. (26) found that participants oftentimes skipped EMA surveys in situations when it was considered inappropriate to respond such as during a conversation or a church service, while Wu et al. (27) showed that surveys were most often not completed in noisier situations containing speech, in which directional microphones and noise reduction algorithms are typically enabled (27). As noted by Wu et al., this will lead to biases in interpretation of subjective EMA reports.

An approach that is unobtrusive and minimizes user burden is to use ongoing remote data logging in which data collected by the hearing aids are automatically and continuously transferred from the aids to a smartphone via a Bluetooth connection. Because smartphones usually have ample space for data storage, fine-grained temporal data can be collected for numerous acoustic parameters. Remote data logging has the potential to provide new insights into ways in which hearing aids are being used in real life (11, 28), and provides new opportunities for research and for development. For example, it has been used to establish evidence regarding daily hearing aid usage for public health decision-making (29, 30), to augment EMA with hearing devices with acoustic information (21, 25) and to support development of advanced hearing aid technology (31–34). Further, by combining remotely logged hearing aid data with that collected from fitness trackers it has been shown that heart rate is significantly and continuously moderated by dimensions of the ambient acoustic environment (32). Finally, remote data logging has also been introduced in a commercially available hearing aid for users to track their own hearing aid usage and sound exposure [HearingFitness™, (35)].

Remote logging is in its infancy and as such there are many unknowns about its reliability and validity. For example, for stable flow of data, most remote logging requires a constant Bluetooth connection between the smartphone and the hearing aids. However, Bluetooth connections can be unstable and not all hearing aid users always keep their smartphone close by, which can then lead to data loss. A thorough investigation of the validity and representativeness of remote data logging is therefore needed to validate its use in audiological research and clinical work.

In this study, we investigate the representativeness of remote data logging to understand whether it provides a quantitative account of hearing aid usage and its association with everyday contextual factors, so that in the future, individual deviations from group-level patterns can be identified and used to support patients and hearing care professionals. We compare information obtained through remote data logging with that obtained through intrinsic data logging to assess the extent to which remotely-logged data reflect daily hearing aid on-time and sound exposure. Our analysis leverages an observational and longitudinal dataset from in-market hearing aid users. The dataset has been reported upon and validated elsewhere (32) and similar data are publicly available online (31).

## Methods

### Participants and Ethics

Participants were users of Oticon hearing aids (Oticon A/S, Smørum, Denmark) who had signed up to use the HearingFitness™ feature via the Oticon ON™ remote control app and used it at least once with their hearing aids between May the 15th and September the 30th 2019. The Oticon ON™ remote control app can be used by users of Oticon Opn™ and newer hearing aids and it provides an interface to keep track of battery status, changing listening program, adjusting volume etc. When signing up to use the HearingFitness™ feature via the app, participants gave informed consent for data to be collected, stored, and used for research purposes on aggregated levels. No other action is required by the participants when using the feature. Note that no personal identifiers nor qualitative characteristics (e.g., age, hearing loss) are being collected. However, since the participants represent a random sample of typical hearing aid users, we speculate that 6 in 10 are male, are aged around 74 years based on hearing aid user surveys (36).

No ethical approval is necessary for this study according to Danish National Scientific Ethical Committee (https://www.nvk.dk/forsker/naar-du-anmelder/hvilke-projekter-skal-jeg-anmelde).

### Data

We extracted a convenience sample of remote logging from 64 hearing aid users. The data, stored in the HearingFitness™ database, consist of minute-based timestamped remote data logs of ambient sound pressure levels (SPLs) and signal-to-noise levels (SNRs) estimated from within the hearing aid processing (35). In addition, for each log, the intrinsically accumulated hearing aid on-time (in seconds) since last clinical visit is stored. More details about the dataset can be found in Christensen et al. (32). In all following analyses, only data logged between 6:00 and 00:00 are used. This is done to minimize confounding effects from night-time logging occurring while the hearing aids were not actively worn (e.g., by forgetting to turn off hearing aids at night). In addition, only participants with at least 2 days of remote data logging were included to enable a comparison of remote logging and the intrinsically accumulated hearing aid on-time. After filtering, the data represents bi-lateral hearing aid usage from 62 users across a combined total of 1,099 days. When separated by hearing aid side, the data consist of 2,054 days of usage.

### Pre-processing

Daily hearing aid usage can be estimated from two sources: the data log timestamps obtained through remote datalogging (henceforward referred to as *T*_*remote*_), and the intrinsically accumulated hearing aid on-time (henceforward referred to as *T*_*intrinsic*_). From the remote logs, each timestamp represents 60 s of hearing aid on-time and smartphone connectivity. Thus, *T*_*remote*_ is estimated by counting the number of timestamps within a selected time-window that are longer than 1 min. For example, counting 40 unique timestamps in 1 h equates to 40 min of hearing-aid use in that hour. For data to be saved in a remote log, smartphone connectivity to the hearing aid is required. On the other hand, the intrinsically accumulated hearing aid on-time represents absolute hearing aid on-time regardless of smartphone connectivity. More specifically, from a series of *N* consecutive data logs with accumulated on-time times *t*_1_*, …, t*_*N*_ spanning time-window *T*, the total on-time is estimated as $T_{intrinsic}=\overset{N}{\underset{n=2}{\sum }}t_{n}-t_{n-1}$, which compensates for potential gaps from inter-log times longer than 1 min (e.g., due to momentarily lost Bluetooth connectivity). A comparison of hearing aid usage from the two estimators (*T*_*remote*_ and *T*_*intrinsic*_) within a time-window provides insight into the amount of data lost due to a lack of smartphone connection. Connectivity is thereby defined as the proportion of time a hearing aid is connected to a smartphone for the duration of the inspected on-time.

### Statistical Analysis

#### Clustering of Usage Patterns

We used K-means unsupervised clustering to identify archetype usage patterns, where a usage pattern is defined by the minutes of hearing aid usage per hour during a day from 6:00 to 00:00. Thus, the input data consisted of vectors *X*_*d*_ = {*x*_*i*_; *i* ∈ *N*} with variables *x*_*i*_ defined by usage in minutes, *x*, per hour, *i*, and days *d* = {1, .., *D*} pooled among all participants in the sample. The K-means algorithm then assign each *X*_*d*_ to a cluster centroid so that the Euclidian distances between all *X*_*d*_'s and centroids are minimized (i.e., the total sum of squares distance) while iteratively selecting cluster centroids that minimize the intra-cluster variation (i.e., the within-cluster sum of squares distance). The optimal number of clusters was selected based on the elbow method (37), which selects the number of clusters that lead to only a minor change in the total sum of squares with the addition of more cluster centroids. Finally, the clustering was evaluated with the Silhouette Coefficient (38) assessing how densely clustered each *X*_*d*_ is around the centroids. The Silhouette Coefficient (*SC*) is calculated for each vector *X*_*d*_ as:


$$\begin{array}{l} SC=\frac{b-a}{\max\left(a,b\right)}, \end{array}$$

where *a* is the mean Euclidian distance between a vector and all other vectors in the same cluster, and *b* is the mean Euclidian distance between a vector and all other vectors in the next nearest cluster. In general, the *SC* is bounded between −1 for incorrect clustering and +1 for highly dense clustering. Coefficients around zero indicate overlapping clusters (that is, the distance from a vector to the two clusters is equal) and the coefficient is positive and higher when clusters are dense and well-separated.

The K-means optimization was implemented in R using “cluster” package [version 2.1.0, (39)].

#### Associating Hearing Aid Usage With Ambient Sound Characteristics

The associations between daily hearing aid usage and parameters of the acoustic environment (SPL and SNR) were tested using a linear-mixed model (LMM). LMMs are ideal for regressing longitudinal and hierarchical multi-level data allowing for random offsets and slopes from grouping variables (40). The model included total daily hearing aid usage as the dependent variable in hours. The independent predictors consisted of the daily (logarithmic) mean SPL–i.e., the equivalent continuous mean SPL (SPL L_eq_), daily median SNR, and the daily standard deviation of the SPL (SPLSD). These predictors represent the intensity, the quality, and the loudness diversity of the daily sound exposure. For simplicity, we did not include the daily standard deviation of the SNR because SNR is a relative measure (i.e., a difference between noise and signal) and, thus, daily variations in SNR cannot be easily interpreted.

The random effect's structure accounted for the day of the week, hearing aid laterality, and individual offsets in daily usage (i.e., random intercepts) and individual sensitivity toward acoustic characteristics (i.e., random slopes). Accounting for laterality effectively ensure that coefficients from the LMMs are estimated based on the average ambient sound sensed between the left and right hearing aid, while accounting for individual sensitivity toward acoustic characteristics with random slopes ensure that individual differences in e.g., loudness growths functions, do not affect the results.

Besides inspecting coefficient magnitude and confidence intervals, significance of predictors was assessed by likelihood ratio testing against an intercept-only model. Prior to modeling, extreme outliers in the acoustic characteristics (i.e., values <1% quantile and larger than the 99% quantile) were removed to ensure normality of the residuals. This removed 147 observations (hearing aid usage days) in total.

To assess the degree of multicollinearity within the model, the generalized variance inflation factor (GVIF) was computed using the ‘car' package in R [version 3.0.8, (41)]. The GVIF is a generalization of the variance inflation factor (VIF) that can be applied to categorical explanatory variables (42). Values of GVIF <4 are usually considered to be acceptable (43).

Effects size estimates were computed by separating the explained variance by fixed effects alone or by the full model using the pseudo-R-squared for Generalized Mixed-Effect models implemented in the “MuMIn” package [version 1.43, (44)] in R. LMMs were fitted in R using the “lmerTest” package [version 3.1, (45)].

## Results

### Identifying Hearing Aid Usage Patterns

**Figure 1:**
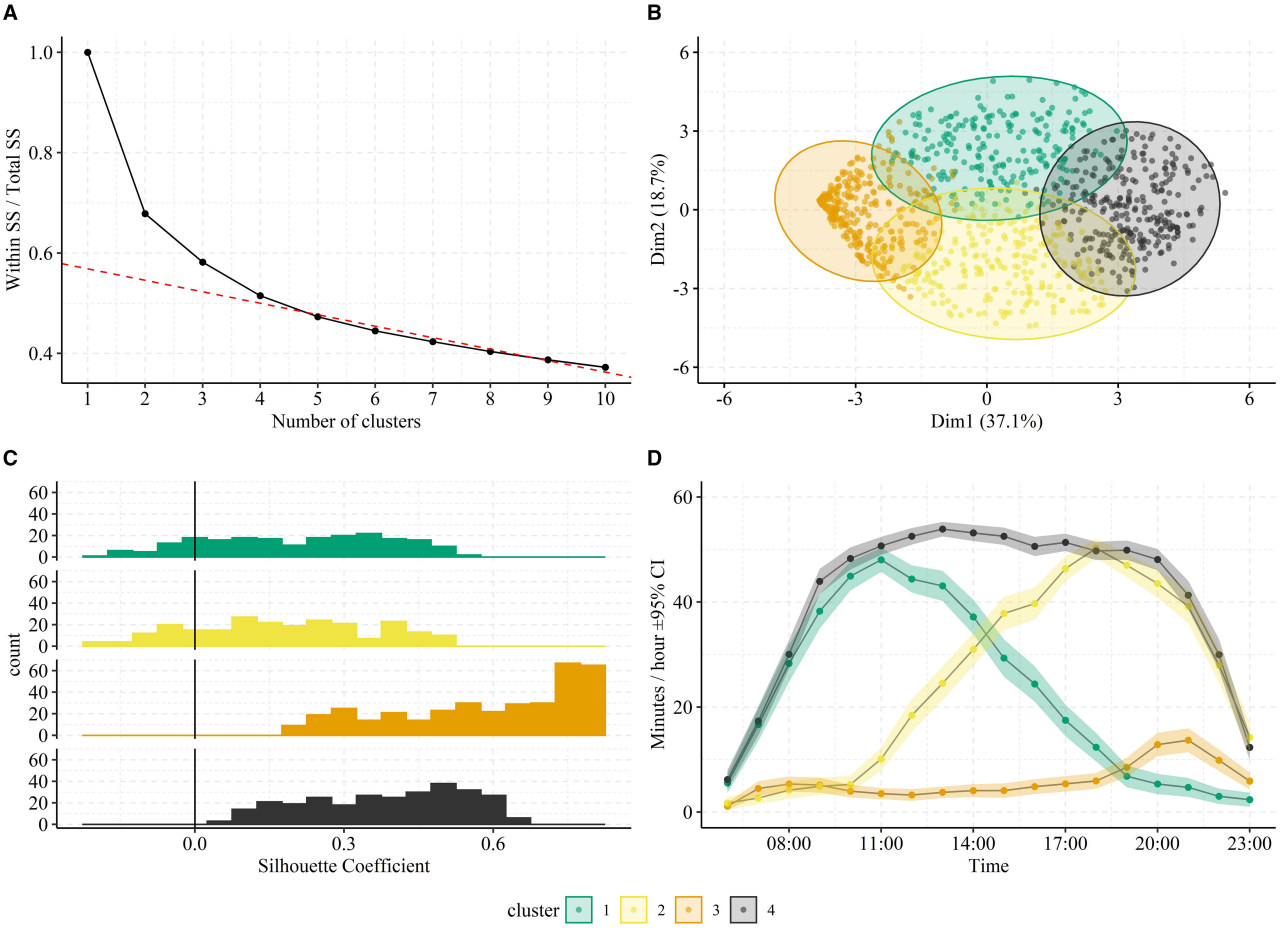
Clustering of pooled hearing aid usage patterns. In **(A)**, the total within-cluster sum of squares (SS) normalized by the total SS is plotted against number of clusters. The “elbow” approach suggests selecting four clusters since adding additional clusters result in minor improvements. This is illustrated by the red dotted line, which represents the best linear fit to >4 clusters. In **(B)**, the two most contributing dimensions of a principal component analysis of the usage patterns are plotted against each other with separation of the four identified clusters (colors). In **(C)**, the average time-course of usage patterns belonging to each cluster is shown. The shaded area in represents the 95% confidence intervals across usage patterns belonging to each cluster. The clusters one to four were associated with 19.1, 21.5, 33.5, and 25.9% of the participants days, respectively. In **(D)**, distributions of Silhouette Coefficients are shown for each cluster. The vertical line at 0.0 represents the threshold for poor clustering (<0).

The fine-grained temporal structure of the data collected by remote logging opens the possibility of examining hourly usage patterns to investigate how hearing aid use varies with e.g., time of day. Here, a K-means clustering algorithm is applied to the pooled hourly usage patterns among all participants and days. Note that we only include usage data from the hearing aid that had the highest daily total. The “elbow” approach, i.e., visual inspection of between-cluster variance vs. cluster number, suggested the existence of four clusters (Figure 1A). These clusters explain 48.5% of the total variance observed between the daily usage patterns in the data. As evident from Figure 1B, the four clusters can be well-separated when inspecting the two most contributing dimensions of a principal component's analysis of the usage patterns. Moreover, the overall mean Silhouette Coefficient (Figure 1C) was 0.37 (*SD* = 0.24) and separated by cluster 1 to 4 it was 0.21 (*SD* = 0.18), 0.18 (*SD* = 0.18), 0.58 (*SD* = 0.18), and 0.39 (*SD* = 0.16). Thus, the Silhouette Coefficients across pooled usage days were predominantly positive indicating successful clustering. Figure 1D shows the average time-course of usage patterns belonging to each cluster, which were associated with 19.1, 21.5, 33.5, and 25.9%, respectively, of the 1,099 days pooled among participants. Cluster 1 indicates a pattern of use predominantly in the morning morning/noon, cluster 2 with predominant use in the afternoon/evening, cluster 3 with low use throughout the day in brief epochs of time, and cluster 4 with constant usage throughout the day.

### Modeling Daily Hearing Aid Usage

Together with usage data, each remote log contains estimates of the ambient acoustic environment. Such data enables investigations of how environmental factors influence hearing aid usage—that is, an examination of true ecological hearing aid use.

**Figure 2:**
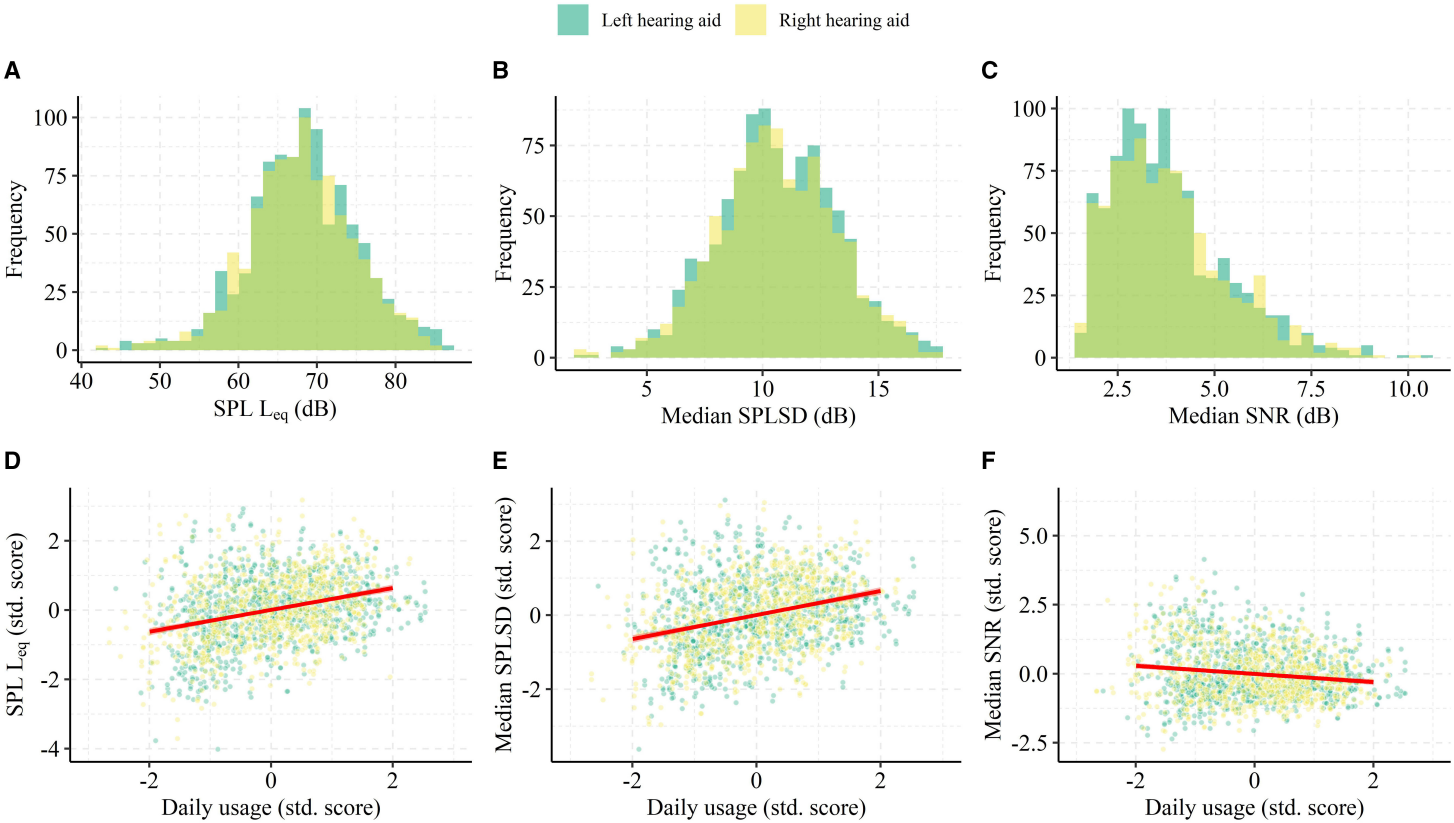
Data for LMM modeling of the association between daily hearing aid usage and the ambient acoustic environment. In **(A–C)**, histograms of daily SPL L_eq_, the standard deviation of the SPL (SPLSD), and the daily median SNR pooled from all participants. In **(D–F)**, scatterplots of the acoustic predictors and the daily usage, both in participant-specific standardized score (i.e., Z-score). For visualization of the trends, the red line represents the best linear fit to data within the interval −2:2 in Z-score.

The daily SPL L_eq_, daily median SNR, and the daily SPLSD were used as predictors to daily total hearing aid usage with usage estimated from remote logging. Figure 2 shows histograms of each acoustic parameter. The SPL L_eq_s and SPLSDs are approximately normally distributed with a grand median of 68.1 dB (*SD* = 6.9 dB) and 10.56 dB (*SD* = 2.6 dB), respectively. The daily median SNRs are right skewed with a grand median of 3.6 dB (*SD* = 1.4 dB), a minimum of 1.6 dB and maximum of 8.6 dB. In fact, only 6.4% of the raw logged SNRs were zero or negative.

The full LMM model (*n* = 1,907 days) explained 35% of the variance in day-to-day total hearing aid usage and of those 15%-point was related to the acoustic predictors alone. In addition, the acoustic predictors significantly improve the model's account of daily total hearing aid usage compared to an intercept-only model [Likelihood ratio test: $\chi_{\left(12\right)}^{2}$ = 229.38, *p* < 0.001] and there are significant main effects associated with each. The fitted coefficients for SPL L_eq_ and SPLSD are positive [β = 0.89, 95% CI = (+0.52 to +1.27), *p* < 0.001; β = 1.30, 95% CI = (+1.00 to +1.60), *p* < 0.001], while SNR had a negative association with daily total usage [β = −0.98, 95% CI = (−1.43 to −0.53), *p* < 0.001]. Day of the week (implemented as a random effect) was significant [Likelihood ratio test: $\chi_{\left(1\right)}^{2}$ = 18.85, *p* < 0.001] and the largest difference in the intercept of total daily hearing aid usage was Monday and Tuesday (+0.65 and +0.45 h) and Friday (−1.21 h). The VIFs were all <1.44. These results suggest that daily total usage is higher on days with more intense and more diverse sound environments, while days with easier listening environments (more positive SNRs) exhibited lower total usage. The lack of covariance between the acoustic predictors indicated by the low VIF suggests that these effects were independent of each other.

### Representativeness of Remote Data Logging

**Figure 3:**
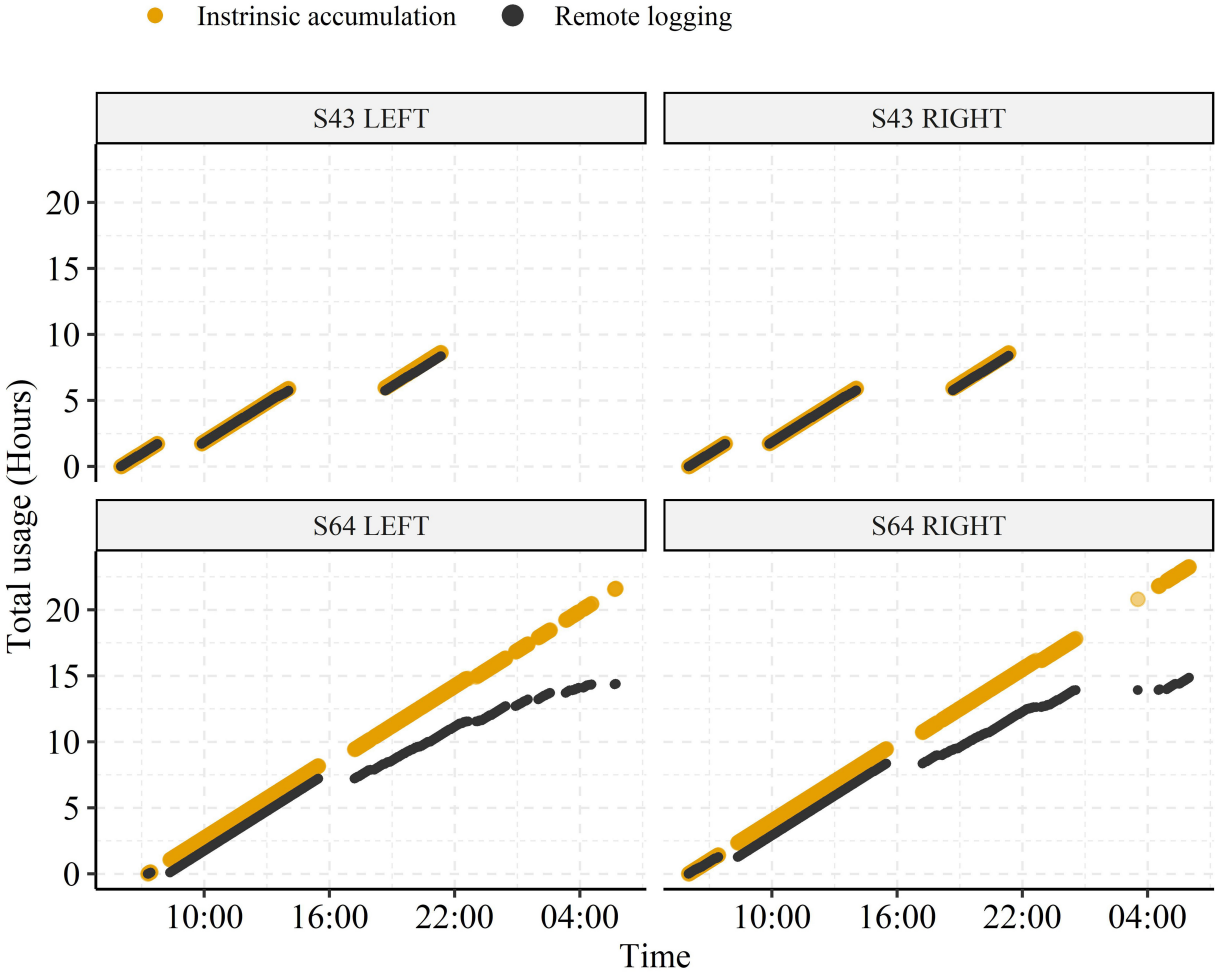
Representative examples of hearing aid usage accumulated throughout the day from two participants (S43 and S64) across 24 h (06:00 to 06:00 next day). The usage is estimated from either intrinsic accumulation (*T*_*intrinsic*_) or from the minute-based remote datalogging (*T*_*remote*_).

While the analysis presented in the preceding sections highlights the potential of using remote logging to understand hearing aid user's ecology, the face-validity and representativeness of such data is still unknown since its generation requires a constant stable Bluetooth connection between hearing aid(s) and a smartphone. Here, we assess the extent to which the minute-based remote data logs correspond to true hearing aid on-time by directly comparing the daily total and the participant-average hearing aid usage measured by the intrinsic accumulation (*T*_*intrinsic*_) with the data log timestamps (*T*_*remote*_). Note that *T*_*intrinsic*_ is not subjected to data loss, thus, it reflects true hearing aid on-time from the first to the last remote log each day. In Figure 3 we plot hearing aid usage accumulated across 24 h (6:00 to 6:00 next day) on a random day for two participants to illustrate differing patterns of connectivity. Participant S43 exhibits a stable connectivity, where the periods without remote data logs correspond closely to times when the hearing aids were turned off. In contrast, in participant S64 the hearing aid usage computed via remote data logs diverge from that obtained through the intrinsically accumulated data from 16:00 onwards. It should thus be clear that the total daily hearing aid usage will differ depending on the estimation used.

**Figure 4:**
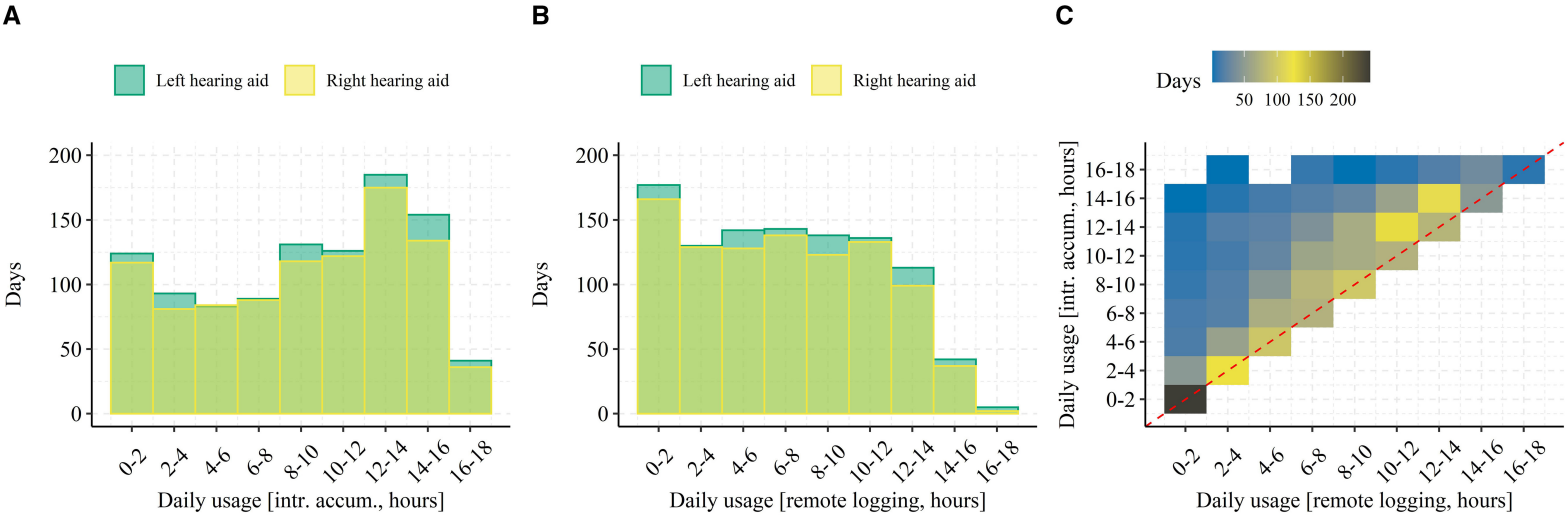
Pooled daily hearing aid usage among all participant days. In **(A)**, a histogram of daily usage estimated from intrinsic accumulation and pooled among all participants, days, and separated by hearing aid side is shown. In **(B)**, the same is shown but for daily usage estimate from remote logs. In **(C)**, the estimated daily usage is shown as a 2D density histogram with the intrinsic accumulation on the y-axis and the connectivity-dependent remote logging on the x-axis. Data falling on the diagonal (red dashed line) represent days with no or <2 h of connectivity issues (i.e., comparable estimation of daily usage from intrinsic accumulation and remote logging).

Pooled across all participants, individual days of data, and hearing aid laterality (totaling 2,054 observations) the median difference (*T*_*remote*_ − *T*_*intrinsic*_) between daily usage estimates is 1.25 h (SD = 2.53 h). Figure 4 shows histograms of daily usage from both estimators (Figures 4A,B, respectively), separated by hearing aid side together with a 2D histogram comparing the daily usage (across hearing aid side) from the two estimators directly (Figure 4C).

**Figure 5:**
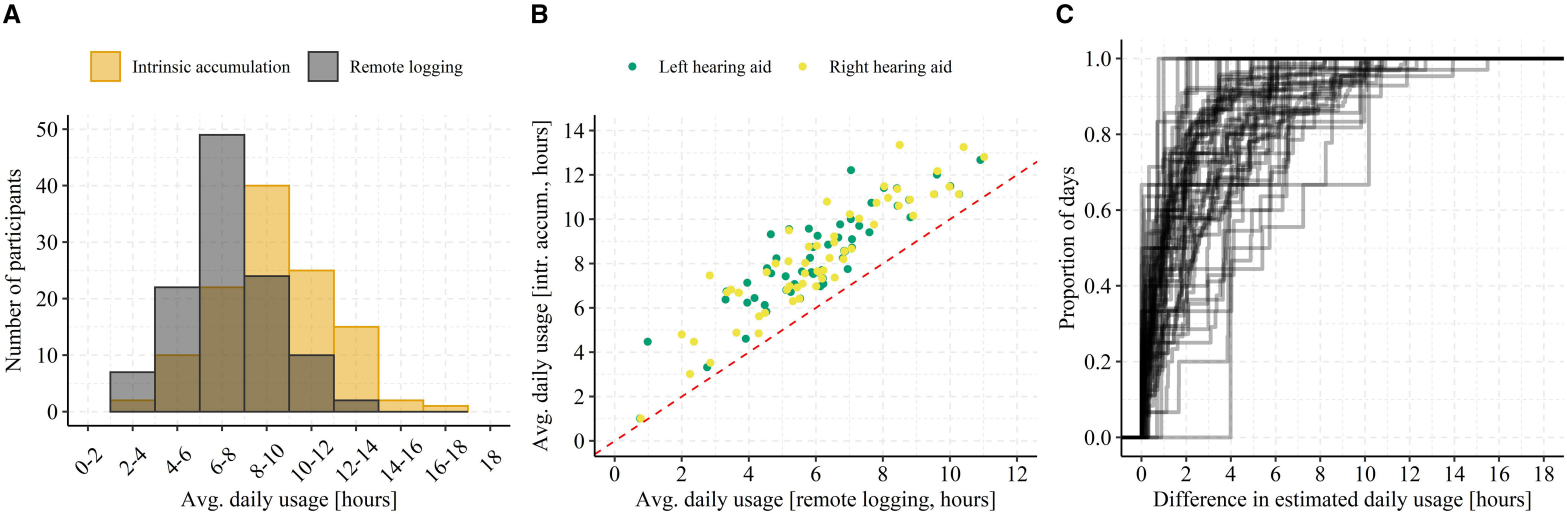
Average daily hearing aid usage. In **(A)**, a histogram of average daily usage (across left and right hearing aid) binned by 2 h intervals and separated by estimation method (i.e., intrinsic accumulation or remote logging). In **(B)**, a scatterplot of average daily usage for both left and right hearing aid from each participant with usage estimated from intrinsic accumulation on the y-axis and from remote logging on the x-axis is shown. The red dashed line represents a y = x relationship. In **(C)**, each trace represents one participant's cumulative distribution function for the daily difference between usage estimates. Note that traces with big jumps (“steps”) on the y-axis are indicative of participants with only few days of data logging.

When averaged across each participants' data and then across all participants (see Figure 5A) the grand median difference in usage estimates (50% quantile in Figure 5C) is 1.59 h (*SD* = 1.26 h). The fact that the pooled median difference in usage estimates is ~0.3 h lower than the grand median difference in usage estimates suggests that some participants consistently exhibit poorer connectivity than others. This might also explain the rather large difference in the two histograms of daily usage (Figures 4A,B)—that is, it might be driven by few participants experiencing many days with poor connectivity. However, from the histogram in Figure 5A, data loss from poor connectivity “left-shifts” the distribution of average daily hearing aid usage to lower values but doesn't otherwise change its shape. In addition, the scatterplot on Figure 5B shows that most participants experience data loss (all points are above the dashed line), that the relative data loss does not depend on average daily usage (points fall parallel to the diagonal dashed line), and that no clear outliers are present. These results suggest that the loss of data in the remote logs due to connectivity is in fact not specific to certain participants but rather it occurs generally. When inspecting the participant-specific cumulative distribution functions (Figure 5C) most participants follow a similar curve, but participants with few days of data logging (identified by traces with large steps on Figure 5C) exhibit either extremely poor or good connectivity.

Finally, when inspecting the distributions of estimated daily usage in Figure 4C, it seems that days with higher hearing aid on-time exhibit a larger difference in usage estimates. That is, loss of remote logs from poor connectivity might be a function of daily hearing aid on-time rather than a constant. Intuitively, this makes sense in that the more time the hearing aids are on, the greater the likelihood that there will be connectivity issues during that time. In Figure 4C, the trend can be seen by the deviation of the mode (i.e., darker yellow squares) of the daily usage from remote logging above the diagonal. This is further corroborated by comparing the histograms in Figures 4A,B. The largest visible difference is occurring between 10 and 16 h of daily usage. To investigate this, the daily difference in usage estimates were computed relative to the proportion of daily hearing aid on-time (Δ*T*_*rel*_) by: $\Delta T_{rel}=\frac{\left(T_{intr}-T_{remote}\right)}{T_{intr}}$, where *T*_*intr*_ is the daily usage from intrinsic logging (i.e., the true hearing aid on-time) and *T*_*remote*_ is the daily usage estimated from remote logs. Next, we computed the average Δ*T*_*rel*_ stratified by participant and hearing aid on-time (from intrinsic accumulation) in discrete bins. If connectivity is a fixed ratio of hearing aid on-time, we would expect a constant Δ*T*_*rel*_ across all bins. On the other hand, if connectivity is a constant, we would expect a declining Δ*T*_*rel*_ with increasing hearing aid on-time.

**Figure 6:**
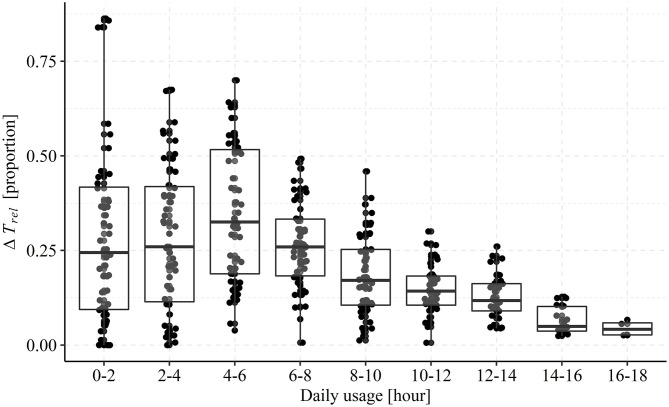
Boxplots and single observations of mean relative difference in usage estimates stratified by the true daily on-time. Each point represents the mean relative difference (Δ*T*_*rel*_) for one participant across all days registered with a total on-time falling into the bins on the x-axis. Each box encapsulates the interquartile range (25th percentile to 75th percentile) and the horizontal line represents the median. The whiskers extend to the largest value within 1.5 times the interquartile range above the 75th percentile (top) and smallest value within 1.5 times the interquartile range below the 25th percentile (bottom).

Figure 6 shows boxplots of Δ*T*_*rel*_ for each bin. Across all bins, the median Δ*T*_*rel*_ is 0.15, which equals an overall connectivity [calculated as 100•(1−Δ*T*_*rel*_)] of 85% of the time a hearing aid is on. However, there is a significant change in Δ*T*_*rel*_ with each step of on-time (LMM regression adjusted by participant, [*F*_(1, 1,776.2)_ = 86.88, *p* < 0.001], suggesting that connectivity first decrease slightly and is lowest between 3 and 7 h of daily usage and then continuously increase. In addition, visual inspection suggests that when on-times are longer than 5 h the inter-individual variability decrease, indicating more stable connectivity patterns across participants. Thus, connectivity is a constant when on-time is >5 h and therefore affect days with higher daily usage less than days with lower daily usage. We also assessed if the connectivity depended on the daily usage patterns identified by clustering (Figure 1). For cluster 1 to 4 the connectivity was 87.01% (*SD* = 23.9%), 85.7% (*SD* = 20.8%), 85.1% (*SD* = 21.9%), and 83.8% (*SD* = 23.0%), respectively.

## Discussion

In this study, we analyzed a longitudinal real-world dataset to demonstrate how remote data logging with hearing aids can produce behavioral and ecological insights into everyday and hourly hearing aid usage. At the same time, we evaluated the validity and representativeness of such data logging by estimating connectivity (i.e., the proportion of time a hearing aid is Bluetooth connected to a smartphone for the duration of the total hearing aid on-time). The data consists of minute-based remote logs collected via Bluetooth transfer of data from hearing aids to smartphones of hearing aid on-time and measures of the ambient acoustic environment sensed by hearing aid microphones.

The K-means algorithm applied to the pooled usage data estimated from remote data logs (Figure 1) identified four distinct clusters of hourly hearing aid usage patterns. Importantly, the Silhouette Coefficients (Figure 1C) demonstrate that the archetypical usage patterns represent an acceptable clustering of the data (i.e., values predominantly above zero). However, there are some days that do not cluster well (especially in cluster 1 and 2) suggesting that, besides the four archetypical patterns, few days exhibit usage patterns that do not categorically fall into one of the four clusters. Interestingly though, the principal component analysis of all patterns (Figure 1B) show only a small degree of overlap among clusters, which suggests that the four patterns occur independently. That is, participants predominantly use their hearing aids according to only one of these patterns on any given day.

Our examination of the relationship between characteristics of the ambient acoustic environment and the daily total hearing aid usage showed significant associations. We saw that days with higher usage were associated with higher ambient sound levels (SPL L_eq_), greater sound diversity (SPLSD), and more difficult listening conditions (lower SNR). These effects can perhaps be attributed to the fact that the longer a hearing aid is worn, the higher is the probability of experiencing varied acoustic environments that include high sound pressure levels and poor signal to noise ratios. However, this might also reflect the fact that individuals choose to wear their hearing aids when their communication needs are greatest, which tends to be in situations in which sound levels are higher and the acoustic environment is more complex, such as at home in the kitchen, in a restaurant, or in meetings (46). Indeed, while past research has had to rely on participant report, data show that active communication often takes place in “noisy” situations. For instance, the participants of Walden et al. (47) reported that 63% of active listening situations involved the presence of noise, Keidser (48) participants reported that 26% of their time was spent talking in quiet with 24% talking when noise was present. Sound recordings show similar. Wagener et al. (49) asked participants to make short recordings of “different situations from your daily life.” They found that about half of the recordings involved conversation, with 11.5% taking place “without background noise,” 18% “with background noise-2 persons,” and 10% “with background noise with more than two persons.” Using EMA and simultaneous sound recordings Wu et al. (50) and Timmer et al. (24) found most EMA reports to be provided for listening situations with low SPLs and high SNRs. However, as noted in the introduction, data collected via EMA are biased toward quieter listening situations because participants often choose not to provide EMA responses when in social situations (26, 27), thus these data neither support nor refute our findings. This also highlights the value of remote data logging to understand the interactions between hearing aid use and the acoustic environment used here. In fact, we argue that given the shortcomings of self-reports and the lack of fine temporal information from intrinsically logged data remote data logging with smartphones using Bluetooth-enabled hearing aids provide a valid way to accurately map daily usage patterns from populations of hearing aid users on a minute-based unobtrusive basis. This is further corroborated by the fact that the representativeness of remote logging is high in the currently examined sample of hearing aid users. We found a median difference of 1.25 h between the true daily hearing aid on-time and the daily usage estimated from the remote data logs when pooled across all 2,054 observations (participants, days, hearing aid side), which corresponds to an overall connectivity of 85%. The absolute difference of 1.25 h seemed to be constant regardless of total hearing aid on-time >5 h (Figure 6), type of usage pattern (Figure 1), and participant-specific average daily hearing aid use (Figure 5B). We assume this difference is due to periods with a lack of smartphone connectivity, which can occur when phone reception is poor, Bluetooth is disabled, or when the hearing aids are out of range of the smartphone. The latter is likely due to the participant not carrying their smartphone with them when using hearing aids. In sum, remotely logged data are a more accurate reflection of hearing aid usage for individuals who wear their hearing aids for longer each day than it is for those who wear their hearing aids for less time. Thus, for detailed and accurate investigations into daily usage patterns, data from days with high connectivity loss should be discarded by limiting analysis of data to individuals who wear their hearing aids for a considerable period of time. Finally, the participant-specific average daily hearing aid usage and the difference between estimators (see Figure 5) suggests that those participants that only contributed with few days of hearing aid data exhibited large inter-individual variability in their connectivity (Figure 5C), but that the average usage across days was not predictive of connectivity (Figure 5B).

### Clinical Relevance

Use of remote data logging potentially has benefits on an individual patient level as well as the population level discussed above. It has the potential to provide deeper insights into an individual's listening lifestyle and how and when they use their hearing aids than has been previously possible. As such, it could then be used by the audiologist to provide counseling at a more fine-grained level than intrinsic data logging permits (16). Perhaps more interestingly and relevant to this paper, at a technological level, the hearing aid could provide automated messages when atypical hearing aid use is detected and/or automatically change hearing aid setting when specific combinations of acoustic parameters and time of day/day of the week are encountered. To “calibrate” these changes to meet patient needs, there could be a process in which there is a period in which patients provide subjective input in order that the hearing aid can “learn” how best to adapt setting to the acoustic environment.

## Limitations

In the current study, we accessed intrinsic logging of hearing aid on-time. However, hearing care professionals can usually also access an intrinsic log of accumulated sound exposure through the clinical computer. It would therefore have been interesting to compare the distributions of the sound environments for the remotely-logged and intrinsically-logged data. However, these data are inaccessible.

Moreover, while we expect the participants in the study to be representative of in-market hearing aid users, we acknowledge that they had all actively signed up for an advanced data tracking feature via the Oticon ON™ remote control smartphone app. Thus, the participants might be more tech-savvy than the average hearing aid user, which might have biased their hearing aid usage patterns.

Lastly, the presented results rely on data from only 62 hearing aid users, which might not be an adequately large sample to generalize insights from. However, the data collected from each participant is rich (minute-based logging), which means that derived insights accurately reflect individual behavior.

## Conclusion

Remote data logging using smartphone-enabled hearing aids can provide rich data regarding hearing aid usage and the ambient acoustic environment in which they are used. The data have high face-validity and smartphone connectivity is generally high (>85%). Days with poor connectivity can be identified and filtered out using statistical methods prior to assessing hearing aid usage patterns and their association to environmental factors.

## Data Availability Statement

There are ethical restrictions on publicly sharing the dataset. The consent given by users did not explicitly detail sharing of the data in any format; this limitation is in keeping with EU General Data Protection Regulation and is imposed by the Research Ethics Committees of the Capital Region of Denmark. Data can be obtained by contacting the corresponding author and signing a non-disclosure agreement.

## Ethics Statement

Ethical review and approval was not required for the study on human participants in accordance with the local legislation and institutional requirements. The patients/participants provided their written informed consent to participate in this study.

## Author Contributions

JC drafted the manuscript and performed that data analysis. GS wrote significant contributions to the manuscript. LH and NP critically reviewed the manuscript. JC, LH, and NP conceptualized the study. All authors contributed to the article and approved the submitted version.

## Author Disclaimer

The views expressed are those of the author(s) and not necessarily those of the NHS, the NIHR or the Department of Health.

## Conflict of Interest

JC, LH, and NP are employed by Oticon A/S. The remaining author declares that the research was conducted in the absence of any commercial or financial relationships that could be construed as a potential conflict of interest.

## Publisher's Note

All claims expressed in this article are solely those of the authors and do not necessarily represent those of their affiliated organizations, or those of the publisher, the editors and the reviewers. Any product that may be evaluated in this article, or claim that may be made by its manufacturer, is not guaranteed or endorsed by the publisher.
